# Highly efficient cell-microbead encapsulation using dielectrophoresis-assisted dual-nanowell array

**DOI:** 10.1093/pnasnexus/pgad155

**Published:** 2023-05-10

**Authors:** Zuyuan Tian, Zhipeng Yuan, Pedro A Duarte, Mohamed Shaheen, Shaoxi Wang, Lacey Haddon, Jie Chen

**Affiliations:** Department of Electrical and Computer Engineering, University of Alberta, 9107 116 Street NW, T6G 1H9 Edmonton, AB, Canada; Department of Electrical and Computer Engineering, University of Alberta, 9107 116 Street NW, T6G 1H9 Edmonton, AB, Canada; Department of Electrical and Computer Engineering, University of Alberta, 9107 116 Street NW, T6G 1H9 Edmonton, AB, Canada; Department of Electrical and Computer Engineering, University of Alberta, 9107 116 Street NW, T6G 1H9 Edmonton, AB, Canada; School of Microelectronics, Northwestern Polytechnical University, 127 Youyi St West, 710129 Xi’an, Shannxi, China; Department of Electrical and Computer Engineering, University of Alberta, 9107 116 Street NW, T6G 1H9 Edmonton, AB, Canada; Department of Electrical and Computer Engineering, University of Alberta, 9107 116 Street NW, T6G 1H9 Edmonton, AB, Canada; Academy for Engineering and Technology, Fudan University, 220 Handan St, 200433 Shanghai, China

**Keywords:** single-cell capture, hydrophilic beads, co-encapsulation, microfluidics, dielectrophoresis

## Abstract

Recent advancements in micro/nanofabrication techniques have led to the development of portable devices for high-throughput single-cell analysis through the isolation of individual target cells, which are then paired with functionalized microbeads. Compared with commercially available benchtop instruments, portable microfluidic devices can be more widely and cost-effectively adopted in single-cell transcriptome and proteome analysis. The sample utilization and cell pairing rate (∼33%) of current stochastic-based cell–bead pairing approaches are fundamentally limited by Poisson statistics. Despite versatile technologies having been proposed to reduce randomness during the cell–bead pairing process in order to statistically beat the Poisson limit, improvement of the overall pairing rate of a single cell to a single bead is typically based on increased operational complexity and extra instability. In this article, we present a dielectrophoresis (DEP)-assisted dual-nanowell array (ddNA) device, which employs an innovative microstructure design and operating process that decouples the bead- and cell-loading processes. Our ddNA design contains thousands of subnanoliter microwell pairs specifically tailored to fit both beads and cells. Interdigitated electrodes (IDEs) are placed below the microwell structure to introduce a DEP force on cells, yielding high single-cell capture and pairing rates. Experimental results with human embryonic kidney cells confirmed the suitability and reproducibility of our design. We achieved a single-bead capture rate of >97% and a cell–bead pairing rate of >75%. We anticipate that our device will enhance the application of single-cell analysis in practical clinical use and academic research.

**Significance Statement**

Microfluidic systems tailored for cell–bead pairing have been applied in single-cell analysis. Major concerns in operating these microfluidic systems include the efficiency of particle loading and cell–bead pairing, which determine the sample utilization and sensitivity of the system. Current designs achieve cell–bead pairing based on stochasticity and thus have low loading efficiency and pairing rates. Here, we demonstrate a microfluidic design called dielectrophoresis (DEP)-assisted dual-nanowell array, which reduces randomness by decoupling bead loading and cell pairing. Incorporation of a dual-well structure and use of a DEP force enable active capture of single cells from suspension. The high cell capture and pairing rate of our device, combined with a simple operation protocol, outperforms other proposed microfluidic systems.

## Introduction

Multiomics at the single-cell level brings an unprecedented in-depth view of numerous biological activities and heterogeneous biogenous components (1–4). With the aid of microfluidic systems, single-cell analysis is possible on a larger scale (>1,000 cells), which significantly reduces the average cost (5). Many microfluidic designs utilize microbeads functionalized with different biomolecules to introduce indexing molecules and the necessary biochemical reactants. For example, streptavidin-coated polystyrene beads coated with capture antibodies have been applied to single-cell protein profiling (6) and fast cell screening based on secreted antibodies (7) or cytokines (8). Recent technologies also use resin or dissolvable hydrogel beads modified with oligonucleotides for single-cell transcriptome analysis to perform high-throughput single-cell RNA sequencing (scRNA-Seq) (9, 10).

Most single-cell microfluidic platforms are constructed with many separate microreaction chambers for every single cell and bead. Mainstream designs include droplet-based systems, nanowell-based systems, and microvalve-based systems. Droplet-based microfluidics systems generate water-in-oil droplets to simultaneously encapsulate cells and beads at high frequency, such as the commercially available 10× single-cell sequencing system (11, 12). In nanowell-based designs, isolated cells/beads are sequentially loaded into each nanowell followed by mechanical or oil sealing (13–15). Valve-based microfluidic systems commonly employ flexible polydimethylsiloxane (PDMS) structures to trap single cells by adjusting the geometry of fluid pathways (16–18). Compared with the droplet-based approach, the nanowell-based method has the advantage of higher portability and suitability for low-input samples (19). In contrast, the droplet-based system has lower fabrication costs and large sample processing volumes (20). Valve-based platforms are well suited to several circumstances, such as size-based sorting of circulating tumor cells (CTCs) combined with single-cell sequencing (21). The most critical step in operating these microfluidic platforms is the pairing between single cells and single beads. Statistically, in conventional stochastic-based pairing approaches, the occupancy of beads and cells in each compartment obeys a double Poisson distribution (22). To reach the optimal cell–bead pairing rate, the concentration of cells and beads should be modulated so that the mean value of each distribution is close to 1. The volume of the microreaction chambers is also limited to holding only a few particles, thus reducing the variance of captured particles in each chamber to attain a sub-Poisson distribution. However, inconsistency in the size of beads and cells makes it difficult to achieve the sub-Poisson distribution for both.

In recent years, many techniques have been proposed to address the issue of single-cell capture and bead pairing in microfluidic systems. For example, by introducing secondary Dean flow with a spiral fluid channel, particle focusing and train formation can be enhanced (23), which decreases the chance of bead multiplets during encapsulation in the droplet-based system (24). Harrington et al. utilized two spiral fluid channels to achieve synchronous trains in bead and cell streamlines, producing reliable particle co-encapsulation. This design achieved a 70% single-cell capture rate, while the percentage of bead doublets was neglected (25). In a nanowell-based design, Bai et al. devised a nanowell transfer approach that employed the dielectrophoresis (DEP) effect to efficiently capture cells which were then transferred into larger bead-loaded wells to enable a double sub-Poisson distribution. Experimental results showed that the overall pairing rate of the nanowell transfer method reached as high as 75%. However, an additional nanowell transfer step in operation was required, and the results were primarily limited by the newly introduced transferring efficiency of about 82% (26). In laboratory use, a nanowall-based design can achieve a high pairing rate by abandoning the microfluidic channel and manually loading the sample. Zhou et al. proposed an open-format hierarchical loading microwell chip that is capable of >70–75% object pairing rate with gravity (27). Very recently, valve-based methods have shown a relatively higher pairing rate (>70%) compared with other approaches (21, 28). However, in addition to the complexity of the operation which requires real-time controlling of microvalves, the difficulty of upscaling and the sensitivity of channel blockage limit their analysis volume (<1,000 cells) and broad range application (29).

Many devices integrated with micro- or nanowell arrays and electrodes devised for diverse single-cell analysis tasks, such as cell–cell interaction, single-cell sorting, intracellular constituent analysis, etc., have already been reported (30–34). The reliability and simplicity in the fabrication of these designs have also been well demonstrated (35, 36). Here, we present a microfluidic platform based on a DEP-assisted dual-nanowell array (ddNA) for efficient cell–bead pairing and co-capsulation. Most current nanowell-based microfluidic devices contain thousands of nanowell units to achieve high-throughput sample analysis. Our microfluidic device was designed to include 3,000 ddNA units in 10 individually operated sectors, to enable the upscaling and downscaling by simply adding or removing sectors without electrothermal interference. In our ddNA unit, the nonsymmetric interdigitated electrodes generating the spatially constrained DEP force were sandwiched by a photoresist dual-well structure and a glass-etched nanowell. The extended depth of the larger bead-trapping well and the DEP force generated under the smaller cell capture well permit a high loading efficiency and pairing rate for beads and cells. By employing a 3D microstructure design, particles of dissimilar sizes could remain in ddNA units under a high flow rate with a low multiplet rate. Here, we report the validation of the ddNA device through experimental results showing the superiority of our design with ∼97% bead occupancy and >75% single-cell pairing rate. The device employs a simple operational procedure, thereby positioning it as a platform technology for single-cell analysis.

## Results and discussion

### Design and operating principle

**Fig. 1.**
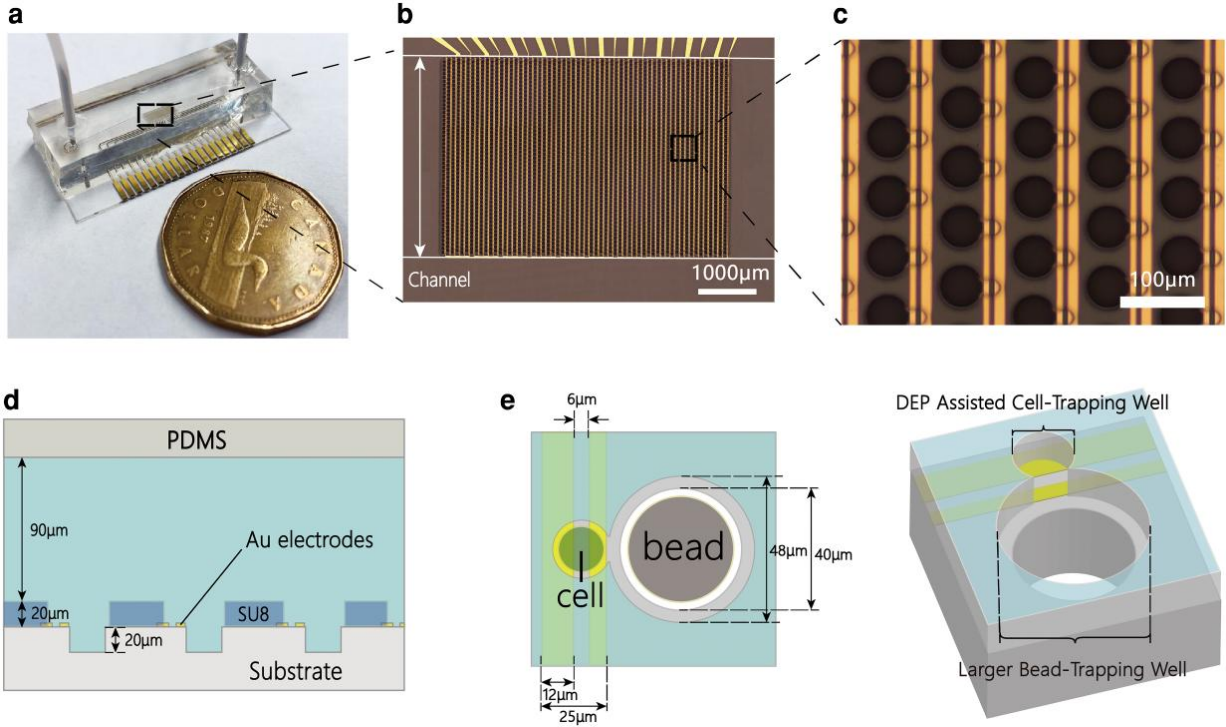
Design and configuration of the ddNA microfluidic device a) assembled ddNA microfluidic device. b) Microscope image of gold electrodes and the dual-nanowell array. The full chip contains 3,000 dual-nanowell structures separated into 10 individually controlled sectors. c) Microscopic close-up image of the nanowells and the nonsymmetric interdigitated electrodes. d) Cross-sectional schematic of the assembled device (not to scale). e) Top view and 3D schematic of the single ddNA unit. Single cells and single hydrophilic beads are captured in an interconnected dual-well structure.

The ddNA microfluidic device (Fig. 1a and b) was designed and fabricated in a four-layer sandwiched structure which included an etched glass layer, a gold electrode layer, an SU8 dual-well layer, and a PDMS channel cover (Fig. 1c and d). The pattern of the SU8 dual-well structure includes two nanowells with different dimensions, 20 and 48 μm in diameter, placed adjacently. The round glass area with a diameter of 40 μm under the larger nanowell was etched for an extended 20 μm depth to fit the dimension of hydrophilic beads (∼35 μm in diameter). The gold electrodes were exposed at the bottom of the smaller well to generate the DEP force for active single-cell capture. By introducing 20 signal pads, interdigitated electrodes were separated into 10 individually addressable sectors forming a multisectorial pattern (Fig. 1b) to reduce potential electrothermal interference and ensure scalability of the whole system (26). The other advantage of this multisectorial design is its applicability to cell sorting in mixed or complex samples, which was described in our previous work (31).

**Fig. 2.**
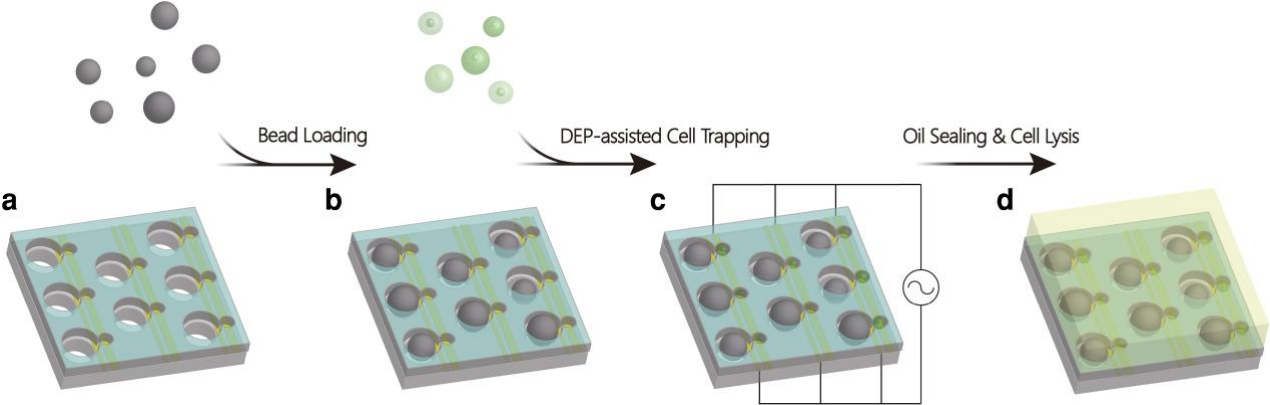
A schematic illustration of the operational procedures of the ddNA device to realize the cell–bead co-encapsulation task. a, b) Hydrophilic beads are loaded into the device to preoccupy the larger trapping wells, and cells then flow into the channel with an AC power source. c) Cells are actively captured in smaller wells by DEP force. d) Fluorinated oil is introduced into the channel to seal the microwell array, and a freeze-thaw cycle lyses the cells in an encapsulated microreaction chamber.

In general, the operational process of the ddNA device involves five steps: chip preparation, bead loading, cell loading, microchamber sealing, and cell lysis, which is comparable with the operation of other proposed nanowell-based microfluidic devices used for cell–bead pairing (6, 14) (Fig. 2). Cells and beads are captured in different parts of the ddNA unit after sample loading (Fig. 1e). The smaller well was designed with a 20 μm diameter to achieve single-cell capture while minimizing the formation of doublets. The larger well region, which is dedicated to capturing beads, was also designed to accommodate only one hydrophilic bead. Since the ddNA unit includes two separate but interconnected components for different-sized particle trapping, our main concern during the design process was addressing the possibility that cells might fall into the larger wells. Therefore, the bead-loading step was set prior to the cell-loading step, so that the larger wells were preoccupied with beads during cell loading.

In our ddNA device, the active single-cell capture mechanism is based on DEP-induced force. The DEP force appears when a particle is more or less polarized than its surrounding media in a nonuniform electric field. The relative intensity of polarization can be mathematically described by the Clausius–Mossotti (CM) factor defining the positive or negative of DEP force, that is, whether a particle is attracted toward or repelled from the region of high electric field strength gradient magnitude (37). The time-averaged DEP force acting on a spherical particle of radius *r* is given by


(1) $$\begin{array}{c} F_{\mathrm{DEP}}=2\pi \varepsilon_{m}r^{3}Re\left\{\frac{\varepsilon_{p}^{*}-\;\varepsilon_{m}^{*}}{\varepsilon_{p}^{*}+2\varepsilon_{m}^{*}}\right\}\nabla {\left|E_{\mathrm{rms}}\right|}^{2} \end{array}$$

where the *E* is the surrounding electric field. The fraction in curly brackets determines the CM factor. $\varepsilon_{p}^{*}$ and $\varepsilon_{m}^{*}$ are the relative complex permittivity of the sphere particle and medium, respectively, each given by $\varepsilon^{*}=\varepsilon +(\sigma /j\omega )$, where ω is the angular velocity of the applied electric field, ε and σ are, respectively, the permittivity and conductivity of the dielectric material and $j=\sqrt{-1}$. The AC nonuniform electric field can be generated by applying a sinusoidal voltage to the interdigitated electrodes.

Analysis of intracellular material requires cells to be lysed after cell pairing. In the operation protocol of our ddNA chip, freeze–thaw was employed to induce lysis of cell membranes since it offers advantages of minimal cross-contamination thereby eliminating the need for rapid fluid exchange (19, 38–40). Once the cells and beads were trapped into ddNA units, the nanowells were sealed with fluorinated oil. The device was then placed in a −80°C fridge or dry ice/ethanol bath and subsequently at room temperature to perform freeze–thaw cycles. The optical transparency of the materials used in device fabrication allowed the experimental procedure to be visually observed.

### Hydrophilic bead loading

To alleviate the potential issue of cell doublets, beads should be loaded first to occupy the larger nanowell in the dual-well microstructure. Given that the beads range from 10 to 20 μm in radius, the diameter of the larger bead-trapping well on the glass etching layer was designed to be 40 μm, while the diameter of the opening on the SU8 layer was slightly larger to have a tolerance for misalignment during fabrication. The bead-loading process included three substeps: perfusing, trapping, and washing. First, beads were suspended at a concentration of 6 to 9 × 10^5^ beads/mL, and 15 μL of bead suspension was pumped into the channel at 8 to 10 μL/min. After perfusing beads across the entire channel in <1 min, the pump was stopped to let beads settle for 40 s for trapping. By moving beads in the channel back and forth through pumping and withdrawing fluid at a relatively lower flow rate (∼4 μL/min), the concentration of beads above the ddNA feature area was maintained at the proper level to ensure high bead occupancy. Unlike droplet-based microfluidic devices where all beads, either paired with cells or not, are mixed, in the nanowell-based design, excess particles are easily recycled via collecting the used washing buffer. Due to size exclusion, no bead multiplets were observed during the experiment.

**Fig. 3.**
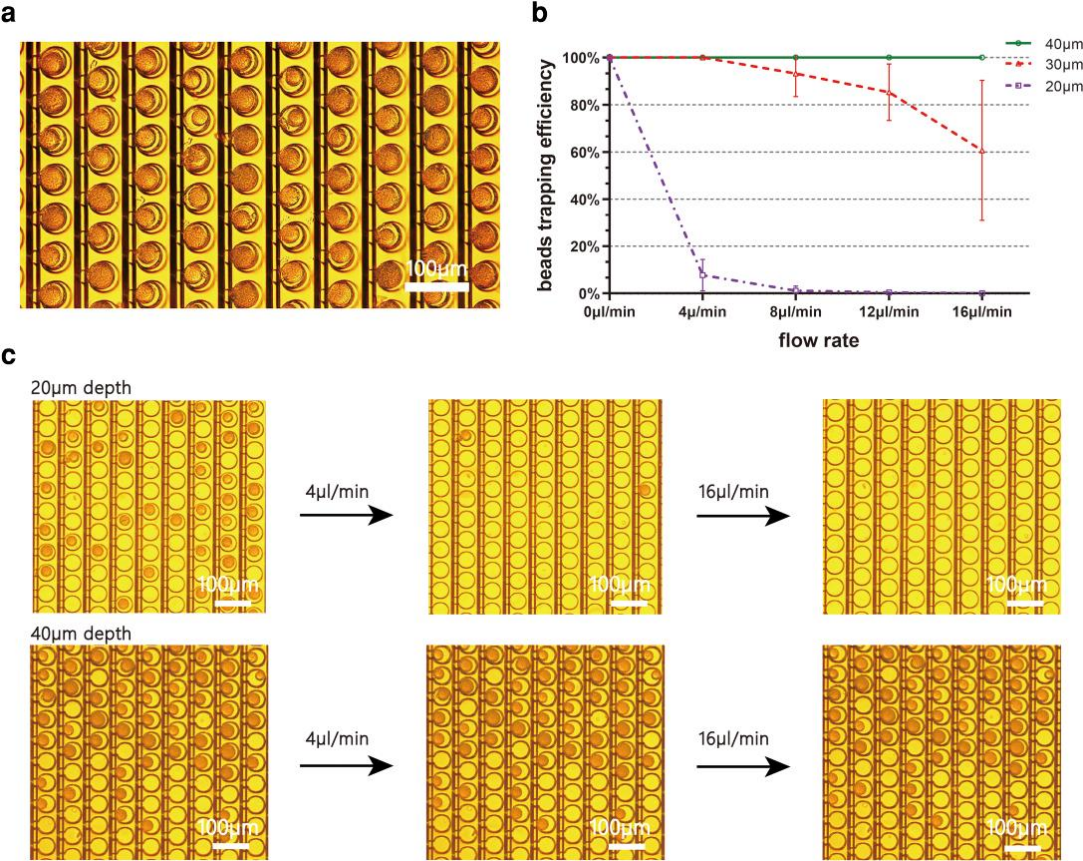
Evaluation of bead-loading occupancy and trapping rate with different designs and flow rates. a) Efficient bead loading achieves >97% occupancy through perfusing, trapping, and washing steps. b) Relationship between bead-trapping efficiency and washing flow rate for different well depths. Each data point shows the mean and standard deviation of the percentage of trapped beads at randomly chosen locations in >200 ddNA units in three independent experiments performed on the same chip. c) Demonstration of beads trapped in 20 and 40 μm depths under different flow rates.

After reaching maximal bead occupancy, extra beads can be washed away by applying a higher flow rate. Due to Stoke’s drag force, the increased flow rate may flush the beads out of the well. Since the drag force is intrinsically induced by the stress acting on the surface of beads, shallower well depth will have more area of bead surface exposed to the high-velocity fluid flow that drastically decreases the bead-trapping rate. Although a deeper well depth can prevent beads from being flushed away in the washing step, the rate of cell multiplets may increase because the cell- and bead-trapping wells are interconnected. Also, a high washing flow rate will efficiently remove beads clusters and prevent cross-contamination during buffer exchange. Therefore, experiments were conducted to investigate the relationship between the nanowell depth and the trapping efficiency to find the minimum depth that could capture the hydrophilic beads at a high flow rate. In these experiments, we achieved different nanowell depths by controlling the thickness of the SU8 layer (Fig. S1). First, beads were perfused and loaded into the 20, 30, and 40 μm depth nanowell arrays as previously described. Next, the flow rate was controlled to increase to 4, 8, 12, and 16 μL/min (Fig. 3b). Since the volume of the fluidic channel was estimated to be <10 μL, the flow rate of 16 μL/min was sufficient to achieve the buffer replacement in <40 s. The bead-trapping efficiency was calculated by Eq. 2, the number of trapped beads at different washing flow rates divided by the number of beads in the nanowell after the initial trapping step.


(2) $$\begin{array}{c} E_{\mathrm{trap}}=\frac{N_{\mathrm{trapped}}}{N_{\mathrm{trapped}}+N_{\mathrm{loss}}}\; \end{array}$$

In the 20 μm depth nanowells, >90% of the beads were flushed away at a flow rate of 4 μL/min. After increasing the microwell depth to 30 μm, the loss of beads significantly decreased. However, on average, >15% of beads were still flushed away in the 30 μm depth wells at a flow rate >10 μL/min. Furthermore, the increased trapping efficiency variation indicated a decrease in the system's robustness, suggesting that the 30 μm depth wells are sensitive to the deviation generated during system fabrication and setup. This could be attributed to the variation in bead diameter, flatness of the SU8 layer, and unstable pressure from the syringe pump. In the 40 μm depth bead-trapping wells, no bead loss was observed in the experiment even at 16 μL/min flow rate. Due to the high trapping efficiency, beads in the channel can be moved back and forth quickly to achieve high occupancy. The experimental results also verified that, with 40 μm depth wells, single beads could be trapped in >97% of the ddNA units (Fig. 3a and Fig. S2). Since the DEP force generated at the bottom of the cell-trapping well is short ranged, DEP-assisted cell capture will be limited with higher microwell depths. Therefore, the glass etching technique was employed in the fabrication to realize the extended depth of the final dual-stage design.

### DEP-assisted single-cell trapping

Conventional nanowell-based microfluidic designs have achieved single-cell loading by diluting cells to a relatively low concentration and relying on gravitational force. However, this approach tends to be less efficient since most of the beads remain unpaired due to the low cell concentration (41, 42). In addition, the effect of gravity force dramatically decreases as the size of an object decreases and a long sedimentation time or an external force, i.e. rocking or centrifugation, is required for cell capture. In contrast, the active capture technique enabled by DEP force allows cells to be captured in a continuous flow. Particles with larger surface areas, such as cell clusters, will undergo higher drag force in the continuous flow and thus are more difficult to capture than single cells. Therefore, applying a constant flow during DEP-assisted cell capture is beneficial for improving the single capture rate. However, the larger drag force induced by the high flow rate may weaken the effect of DEP force and reduce the single-cell pairing rate. Therefore, the relationship between flow velocity and cell pairing rate was investigated in a set of experiments to determine the flow rate which provides the highest single-cell pairing rate. In this case, the pairing rate was calculated by Eq. 3, the number of ddNA units that have single cells paired with single microbeads divided by the number of beads trapped.


(3) $$\begin{array}{c} R_{\mathrm{pair}}=\frac{N_{\mathrm{paired}}}{N_{\mathrm{paired}}+N_{\mathrm{non}\text{-}\mathrm{paired}}} \end{array}$$

**Fig. 4.**
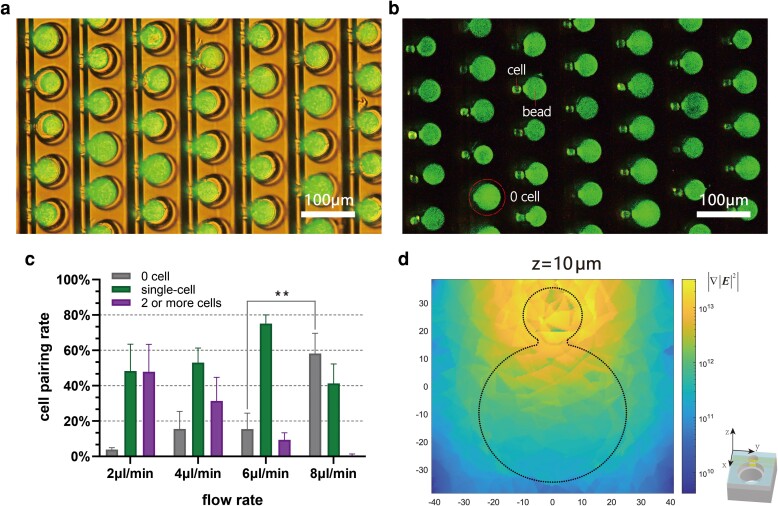
Evaluation of DEP-assisted cell pairing, pairing rate, and the spatial magnitude distribution of DEP force. a) Cell–bead co-capture in ddNA units. b) Fluorescent image of the co-captured cell–bead pairs. Most of the beads were paired with a single cell, enabling higher bead utilization compared with other microfluidic systems. c) Relationship between the flow rate and single-cell pairing rate in a continuous flow cell-loading manner. Each bar shows the mean and standard deviation of the three experiments repeated on different sectors of the same chip. More than half of the ddNA units chosen at random locations were counted. d) Numerical simulation of the magnitude of $\nabla |E{|}^{2}$ vector at the *z* = 10 μm plane above a ddNA unit.

After excess beads were washed away, human embryonic kidney (HEK) 293 cells suspended in low conductive DEP buffer at a concentration of about 2 × 10^6^ cells/mL were loaded into the channel at different flow rates with electrodes connected to a sinusoidal signal source. Since the dielectric property of HEK cells has been well studied, the amplitude and frequency of the electrical signal were selected at 8 Vpp and 1 MHz to generate a DEP force that was strong enough to capture the cells with negligible impact on cell viability (43, 44). The pairing rate in 6 min under the different cell-loading flow rates of 2, 4, 6, and 8 μL/min was tested (Fig. 4c and Fig. S3). A lower flow rate allowed cells a longer time to transfer to the bottom fluid layer and to be affected by DEP force. Therefore, we observed that ∼50% of cells formed multiplets with a flow rate of 2 μL/min. After increasing the flow rate to 4 μL/min, the multiplet rate decreased to around 30%, while the percentage of vacant wells was slightly increased. The optimal single-cell pairing rate was obtained by further increasing the flow rate to 6 μL/min, where a 75% percent single-cell pairing rate was observed. At an 8 μL/min flow rate, on average, >55% of cell trapping wells remained vacant after a 6 min loading period, which is significantly increased compared with a 6 μL/min flow (*P* = 0.0016, one-way ANOVA). Hence, the optimal continuous flow rate for cell loading was estimated to be around 6 μL/min (Fig. 4c and Fig. S4). To further demonstrate the effectiveness of the DEP trapping method, we compared the performance of two neighboring sectors that were either activated or not activated by the DEP signal. The results showed that after the same capture time period, the sector activated by the DEP signal exhibited a significantly higher cell pairing rate than the sector not activated by the DEP signal (Fig. S5).

In the ddNA design, we utilized short-ranged DEP force balanced with the drag force to obtain a desirable single-cell pairing rate. Electrode pairs used for generating the nonuniform electric field were designed in a nonsymmetric pattern, with each of the two electrodes having a different width. This design constrained the electric field intensity peak within the smaller cell-trapping nanowell region. Numerical simulations using commercially available software (COMSOL Multiphysics) were carried out to verify the rationality of the design (Fig. 4d). Equation (1) shows that DEP force is directly proportional to the gradient of the magnitude of squared electric field intensity ($\nabla |E{|}^{2}$). We examined the distribution of the magnitude of $\nabla |E{|}^{2}$ at different heights above a ddNA unit with color maps (Fig. S6). The results indicated that DEP force decreases exponentially in all directions, which suggests that only the cell captured at the center of the DEP-affected region will remain captured at a high flow rate. The decrease of DEP force along the *z*-axis also explains why the cell-trapping well needs to be relatively shallower to effectuate the DEP-assisted cell capture. In addition, we investigated the influence of the IDE finger gap on the electric field gradient. Simulation results showed that the change in the effective electric field gradient with different electrode gaps remains limited (Fig. S7). Thus, adjusting the flow rate which has a linear relationship with drag force is more efficient compared with balancing the DEP and drag force by using different electrode designs.

### Encapsulated cell lysis and visual confirmation

**Fig. 5.**
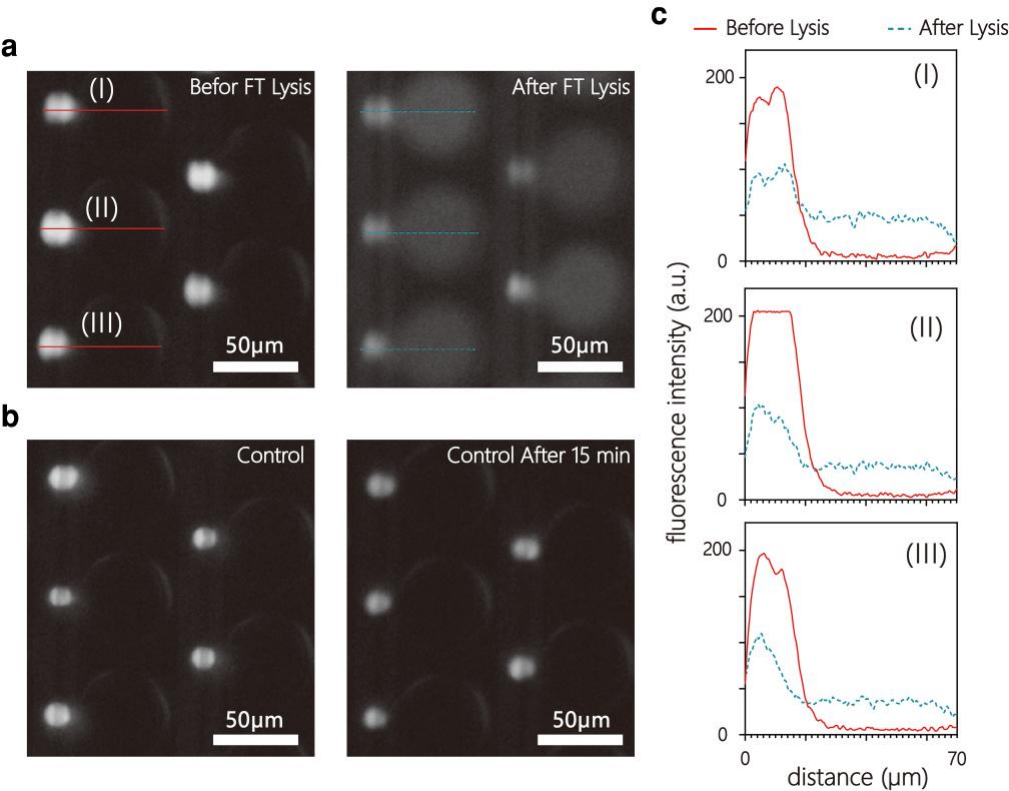
Cell lysis inside enclosed ddNA units. a) Fluorescent image of captured cells in ddNA units before and after (7× exposure) a 10 min freeze-thaw cycle. The dual-nanowell structures have been sealed by the fluorinated oil. b) Fluorescent image of captured cells in the control experiment before and after 15 min at room temperature. A change in cell viability was not observed. c) Change in radial fluorescence intensity due to cell lysis. Fluorescence intensity values were obtained along the solid line and dashed line in image a).

In single-cell analysis, captured cells should be encapsulated in individual reaction chambers to prevent cross-contamination during cell lysis. Oil sealing and pressure sealing are techniques commonly employed to construct the microreaction chamber by closing the opening of each nanowell (14, 45). In our study, the oil sealing method was selected for its ease of use and excellent sealing efficiency (46). Once the desired cell pairing rate was reached, the oil was then pumped into the channel. A visible oil-aqueous interface confirmed that a seal was obtained. As described, freeze–thaw, a contactless cell lysis approach, was applied in our ddNA device to prevent potential cross-contamination among nanowells during the buffer exchange. To determine successful cell lysis and sealing, the diffusion of intracellular fluorescence across the dual-well microstructure (Fig. 5a) was evaluated by the radial distribution of the fluorescence intensity in the sealed ddNA units (Fig. 5c). Containment of intracellular material from single cells was confirmed since there was no leakage of the fluorescent dye out of the closed nanowell, which would result in homogeneous fluorescence intensity across the system. We also conducted a control experiment to verify the effectiveness of the freeze–thaw method. A microfluidic chip with captured cells was placed at room temperature for 15 min, and no fluorescence leakage was noticed (Fig. 5b). These results demonstrate that the oil sealing and freeze–thaw approaches are fully compatible with our device, and demonstrate the applicability of our ddNA device for high-throughput single-cell analysis.

### Potential future enhancements

Our primary goal is to address the limitation of Poisson statistics during microfluidic cell–bead co-encapsulation for single-cell analysis. While many current methods are either limited by sample utilization or complex operating protocols, our novel ddNAs design employs a DEP-assisted active particle capture mechanism and a double-stage microstructure to achieve a high bead utilization and cell pairing rate. We envision our microfluidic device may be readily applied to multiple single-cell analysis tasks and reduce the average cost for single-cell analyses. Particularly, our device is suitable for sorting individual cells to perform single-cell transcriptome analysis (14, 28, 47), which demands the same microfluidic functions as we have reported here. This process involves pairing beads containing short oligonucleotides on the surface with single cells in each paired-microwell to capture the mRNA molecules released from individual cells. The gene library prepared from these beads allows for studying the gene expression profiles of thousands of individual cells, thus helping researchers to identify sporadic cells with altered function (like cancer cells). Our device may also be useful in single-cell cytokine and antibody analysis, where beads coated with antibodies will bind to the specific biomolecules secreted from cells after cell–bead pairing and induce a fluorescent signal on the beads to report the immunochemical reactions (7, 27). As a demonstration of an application, the microfluidic device was used to compartmentalize single cells from a mixed cell population in individual microreaction chambers (in each well). The demonstration emphasizes our ddNA device's applicability for studies that require the isolation of cells at the single-cell level (Fig. S8).

The ability to be easily upscaled suggests that our device may be comparable with commercial products that typically handle tens of thousands of cells. Since DEP force depends on the dielectric property of cells, the selective capture of a target cell type from a mixed sample with this label-free approach is possible (31, 34, 48). Our microfluidic device is expected to integrate cell sorting and analysis functions into one chip, which is promising for many applications, including the analysis of CTCs (21), immunocytes (49), and cell–cell complexes (50) from peripheral blood samples.

While our protocol involves a freeze–thaw method to lyse the cells, many other techniques are compatible with our ddNA device. For example, detergent-based lysis buffers commonly employed in nanowell-based microfluidic chips, and electroporation methods used in many single-cell analyses (51, 52) are expected to be compatible with our device. Each lysis approach has its advantages and limitations (53, 54). We plan to investigate and compare the performance of different lysis methods to expand the potential applications for our platform.

## Conclusion

Microfluidic chips intended to pair single cells with functional hydrophilic beads play an important role in high-throughput single-cell analysis. Hence, developing a microfluidic platform featuring efficient sample utilization and appropriate biomolecule transferability is of crucial importance. Our ddNA device fulfills these criteria via a specific microstructure design without requiring complex operational procedures. Our design allows high sample occupancy and a high single-cell pairing rate by decoupling the particle loading processes and using a DEP-assisted cell capture mechanism. Although DEP capture techniques introduce additional complexity to device fabrication, we believe the benefits of eliminating the need for external control during sample loading outweigh these disadvantages, as suggested in our comparison with other devices showing similar performance. Also, on-chip nanowell sealing and release of intracellular molecules were successfully demonstrated. Based on the results of our studies, we believe our design will be an integral component to our microfluidic platform for high-throughput single-cell analysis purposes.

## Materials and methods

### Chip fabrication

The ddNA microfluidic chip was fabricated utilizing standard photolithography and etching techniques on a 500 μm thick 4 inch quartz wafer. The wafer was first cleaned with hot piranha solution (350°C, 3:1, H_2_SO_4_:H_2_O_2_) for 15 min followed by a deionized (DI) water rinse and N_2_ stream drying. 10 nm Cr and 100 nm Au were then sputtered on the top of the wafer to form the conductive layer. Interdigitated electrodes were patterned using AZ1512 (EMD Performance Materials Corp.) positive photoresist, which was spread at 500 rpm for 10 s and then increased to 5,000 rpm for 40 s to provide ∼2 μm thickness. Next, the photoresist was exposed at 110 mJ/cm^2^ with a UV mask aligner (ABM-USA Inc.) and developed in AZ400K 1:4 developer for 45 s (EMD Performance Materials Corp.). The interdigitated electrode layer pattern was transferred using wet etching techniques with the standard gold etchant (1:4:40, I_2_:KI:H_2_O) and standard chromium etchant (Sigma-Aldrich Inc.), followed by the photoresist stripping with acetone and isopropyl alcohol. Epoxy-based KMPR 1025 negative tone photoresist (Kayaku Advanced Materials Inc.) was utilized as the mask for glass etching patterned with the second photomask. KMPR was spread on top of the wafer at 500 rpm for 15 s and then accelerated to 1,800 rpm and spun for 30 s to form a ∼50 μm thick layer. Before UV exposure at 1,055 mJ/cm^2^, the substrate was soft baked on a leveled hotplate at 100°C for 15 min. Subsequently, the exposed wafer was post baked at 100°C for 4 min and developed in SU8 developer (Kayaku Advanced Materials Inc.) with agitation for 3 min. The wafer with a patterned KMPR mask was then etched with inductively coupled plasma-reactive ion etching (ICP-RIE) equipment (Oxford Plasma Pro 100 Cobra) for 200 min to obtain about 25 μm etching depth. Different etching depths can be obtained by linearly adjusting the etching time. The etching process was performed under 50 sccm CHF3, 10 sccm SF6, and 25 sccm Ar performer with 1,000 W ICP power. Afterward, KMPR 1025 was stripped by immersing the wafer in the cold piranha solution (50°C, 3:1, H_2_SO_4_:H_2_O_2_) for 30 min. The SU8 2010 negative photoresist was employed to form a ∼20 μm thick dual-well structure. SU8 2010 was spread on top of the wafer at 500 rpm for 15 s and spun at 1,000 rpm for 30 s. After soft baking at 65°C for 2 min and 95°C for 4 min, the substrate coated with SU8 was exposed with the third photomask at 150 mJ/cm^2^ and post baked for 2 and 4 min at 65 and 95°C, respectively. Finally, the wafer was developed in the SU8 developer for 1 min before dicing (Disco 3240 Dicing Saw). Additional substrates with 30 and 40 μm thickness of SU8 layers were also fabricated.

The microfluidic channel was fabricated using the soft-lithography technique (55). A master mold for PDMS was fabricated with 90 μm thick SU8 2025 on a 4 inch prime silicon wafer. This thickness was achieved by spreading the SU8 2025 at 500 rpm for 10 s followed by spinning at 1,000 rpm for 30 s. The substrate coated with photoresist was soft baked at 65°C for 3 min and 95°C for 7 min, followed by exposure at 190 mJ/cm^2^ UV light. The post-exposure bake procedure also involved 65 and 95°C baking for 2 and 6 min, respectively. After a 3 min development in SU8 developer, PDMS with a 10:1 mass ratio between base and the curing agent (Sylgard 184 silicone elastomer kit) was poured on the master mold and cured in an oven at 100°C for 30 min. The polymerized PDMS was then peeled off and cut into fittable sizes before inlet/outlet ports were created with a disposable biopsy punch (Robbins Instruments Inc.).

### Device bonding and assembly

The PDMS channel was silanized to create permanent covalent bonding between the channel structure and the SU8-coated glass chip. Silanization was achieved by first treating and activating the PDMS channel with oxygen plasma in a reactive-ion etching machine (Trion Technology, Inc.). The feature side of PDMS was then immersed in a liquid solution containing 99% (3-aminopropyl)triethoxysilane for 45 s, followed by rinsing in DI water and nitrogen stream drying. Subsequently, the PDMS microchannel was carefully aligned and brought into full contact with the glass chip. The complete sealing of the channel structure was ensured by placing the device on a hotplate with 0.5 kg standard calibration weight applied on top and baking at 150°C for 60 min. Finally, the inlet and outlet ports were connected to PTFE tubing (1/16″, Elveflow Microfluidics) with 21G stainless steel connectors.

### DEP buffer

The requisite condition to generate positive DEP force is that the media should be less polarizable than the particle (56). Cell culture media, which typically have a high conductivity and are more polarizable than mammalian cells, are not suitable for positive DEP-induced cell trapping. To facilitate the capture in our ddNA device, cells were resuspended in a sterile-filtered low-conductivity buffer composed of 10 mM HEPES, 3 mM NaOH, 285 mM sucrose, and 1.5 mM MgCl_2_. In addition, the DEP buffer was supplemented with 2% w/v bovine serum albumin (BSA) to block nonspecific cell adhesion. This buffer has been previously used in electroporation studies (57), and the conductivity of the buffer was measured with a conductivity meter (Oakton CON 6+) before each experiment showing an average read of ∼450 μS/cm. The viability of cells in this buffer has also been previously demonstrated (57). All the chemicals used were of analytical grade and purchased from Sigma-Aldrich.

### Cells and beads preparation

Human embryonic kidney cells (HEK-293, ATCC: CRL-1573) were used in this study. Cells were cultured from frozen stock (liquid nitrogen, −196°C) in high glucose Dulbecco's modified eagle medium with l-glutamine and phenol indicator (DMEM, high glucose; Gibco) supplemented with 10% (v/v) fetal bovine serum (SIGMA) and 1% penicillin-streptomycin (Sigma-Aldrich) in culture dishes. Cells were incubated in a humidified incubator at 37°C in an atmosphere of 5% CO_2_. Before each experiment, cells were detached from the culture dish by incubation in 0.05% trypsin-EDTA at 37°C for 5 min and resuspended in 3 mL culture media. Cell concentration was determined by counting with a hemocytometer. Afterward, the cell suspensions were transferred to a centrifuge flask, followed by replacing culture media with low-conductivity DEP buffer to obtain a final concentration of about 2 × 10^6^ cells/mL. The prepared cell sample was stained with Acridine Orange (Invitrogen), Calcein AM (Invitrogen), or DAPI (Invitrogen) following the manufacturers’ standard staining protocols for fluorescent imaging.

The same rigid resin beads (Toyopearal HW-65S, Tosoh Bioscience) used in the single-cell RNA-sequencing workflow (Drop-Seq) were used in our studies. Since beads were loaded before cells, microbeads were also suspended in the DEP buffer prior to the experiment.

### Instrumentation and experimental operation

A custom-made chip holder (Fig. S9) featuring spring-loaded pogo-pins (Mill-Max Mfg. Crop.) was used to electrically connect the microfluidic device to the function generator (Rigol DG822) through a 10× bipolar amplifier (Tabor Electronics 9250) used for signal generation with the desired amplitude. The custom holder was equipped with a set of switches to control the electrical connection of each sector. The generated signal was monitored with a digital oscilloscope (Tektronix TDS 2012B). The ddNA device with the holder was placed on a viewing stage of an upright fluorescence microscope (Amscope FM820TMF143) integrated with a CCD camera (Sony ICX825ALA) for imaging during the experiment. The flow of the beads, cell suspensions, and buffers through the microfluidic channel was precisely controlled with a syringe pump (New Era Pump System).

Before sample loading, the fluid channel of the ddNA device was primed by injecting 500 μL of ethanol in 1 min to eliminate all air bubbles from within the nanowell structures. Thereafter, the device was filled with 5% (w/v) BSA in 1× PBS, followed by incubation at room temperature for 30 min to prevent nonspecific binding. The device was then washed with DEP buffer to remove residual PBS solution in the channel. Beads were then loaded into the channel following the three-step method (perfusing, trapping, and washing) described earlier. The cell suspension was then loaded into the channel with the function generator turned on. After the desired single-cell pairing rate was reached, more DEP buffer was added to wash away excess cells at a flow rate of 10 μL/min. Next, 40 μL fluorinated oil (Fluorinert FC40) was pumped into the device to seal each dual-well structure, forming separate reaction chambers. The device was then placed in a −80°C fridge for 5 min, followed by 5 min at room temperature to lyse cell membranes. Multiple freeze–thaw cycles may be conducted to ensure complete cell lysis. Beads in the microwell can be easily retrieved by flipping the chip and manually washing the fluid channel with a syringe at a high-speed fluid flow.

## Supplementary Material

pgad155_Supplementary_Data

## Data Availability

All data are available within the paper and in Supplementary material.
