# Supplementary material for: Highly efficient cell-microbead encapsulation using dielectrophoresis-assisted dual-nanowell array
Source: PNAS Nexus. 2023 May 10;2(5):pgad155. doi: 10.1093/pnasnexus/pgad155 (PMC10210622; doi:10.1093/pnasnexus/pgad155)
Supplement: pgad155_Supplementary_Data [file pgad155_supplementary_data.docx]

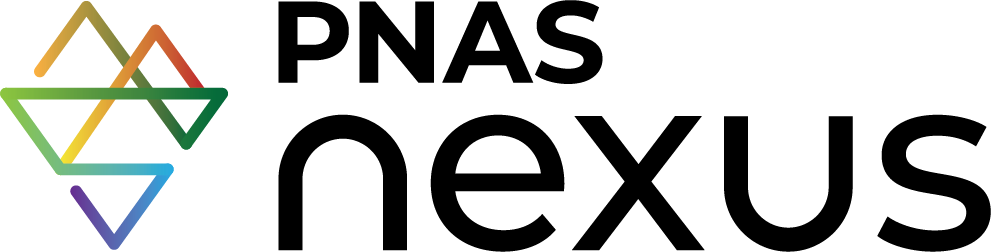


**Supporting Information for**

Highly Efficient Cell-Microbead Encapsulation Using Dielectrophoresis-Assisted Dual-Nanowell Array

*Zuyuan Tian* ***^a^****, Zhipeng Yuan* ***^a^****, Pedro A. Duarte* ***^a^****, Mohamed Shaheen* ***^a^****, Shaoxi Wang* ***^b^****, Lacey Haddon* ***^a^*** *and Jie Chen* ***^a,c^******

**^a^** Department of Electrical and Computer Engineering, University of Alberta, Edmonton, AB T6G 1H9, Canada.

**^b^** School of Microelectronics, Northwestern Polytechnical University, Xi'an, Shannxi 710129, China.

**^c^** Academy for Engineering & Technology, Fudan University, Shanghai 200433, China.

* Corresponding Author: Jie Chen

**Email:**  jc65@ualberta.ca

**This PDF file includes:**

Figures S1 to S9


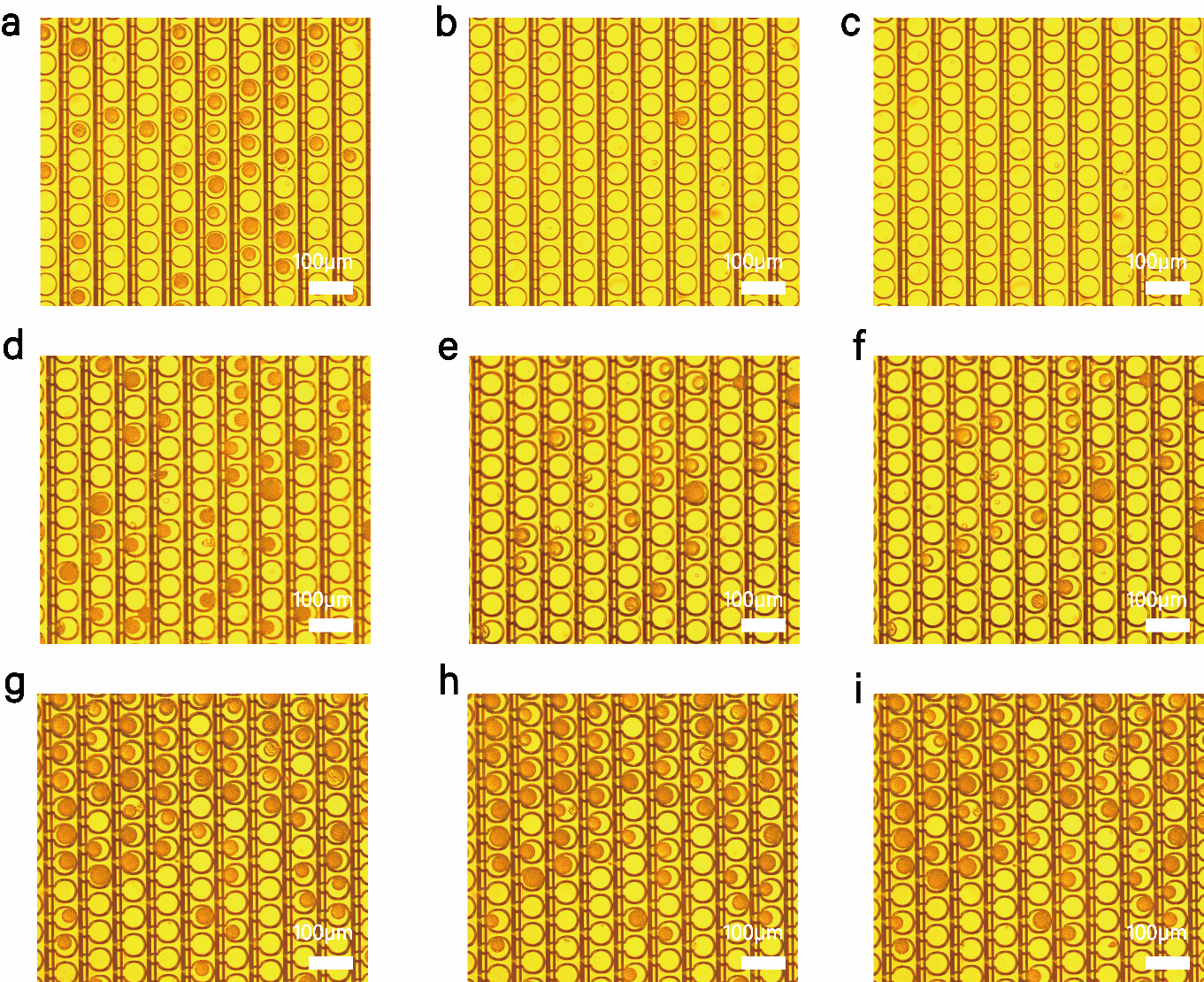


**Figure S1.** Bead trapping at different flow rates. Beads in microwells of 20 μm depth: (a) in a static fluid environment; (b) at 4 μL/min flow rate; (c) at 12 μL/min flow rate. Beads in microwells of 30 μm depth: (d) at 4 μL/min flow rate; (e) at 12 μL/min flow rate; (f) at 16 μl/min flow rate. Beads in microwells of 40 μm depth: (g) at 4 μL/min flow rate; (h) at 12 μL/min; (i) at 16 μL/min flow rate. Bead occupancy is not considered in this experiment.


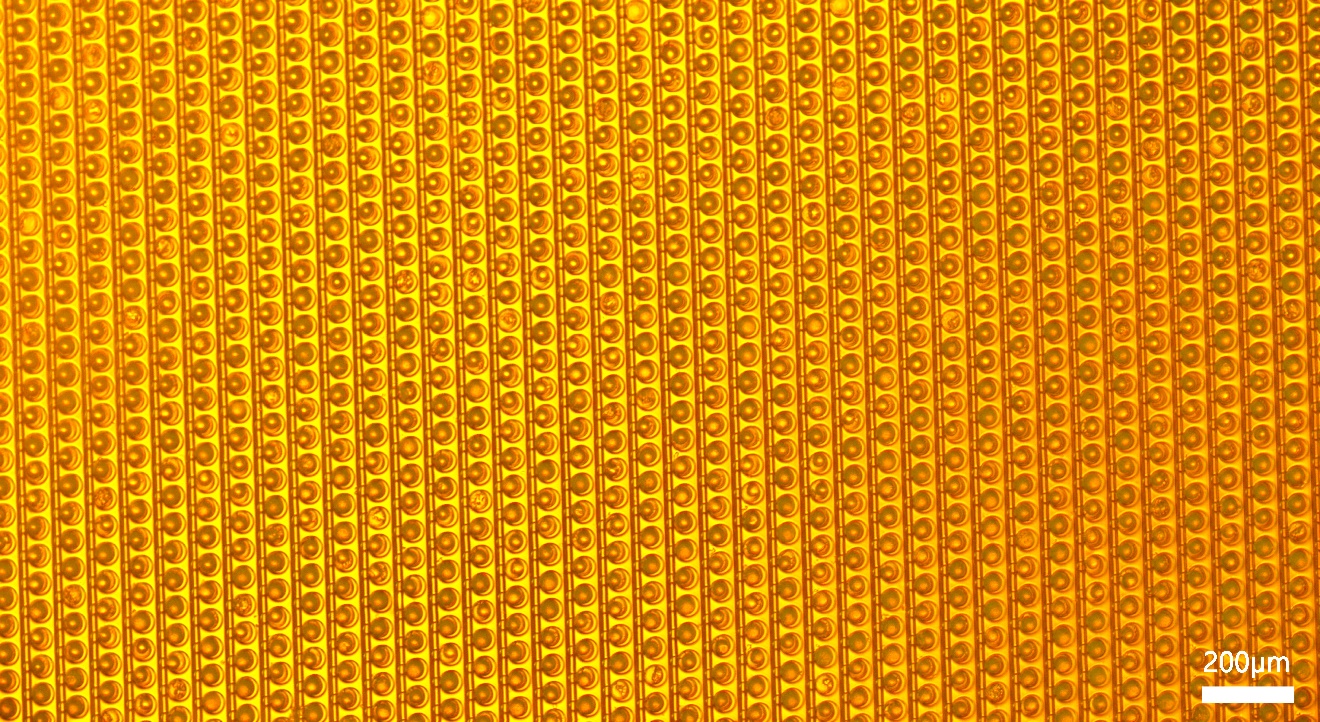


**Figure S2.** The 40 μm depth bead-trapping well formed by 20 μm SU8 and 20 μm glass etching can achieve bead occupancy as high as 97%.


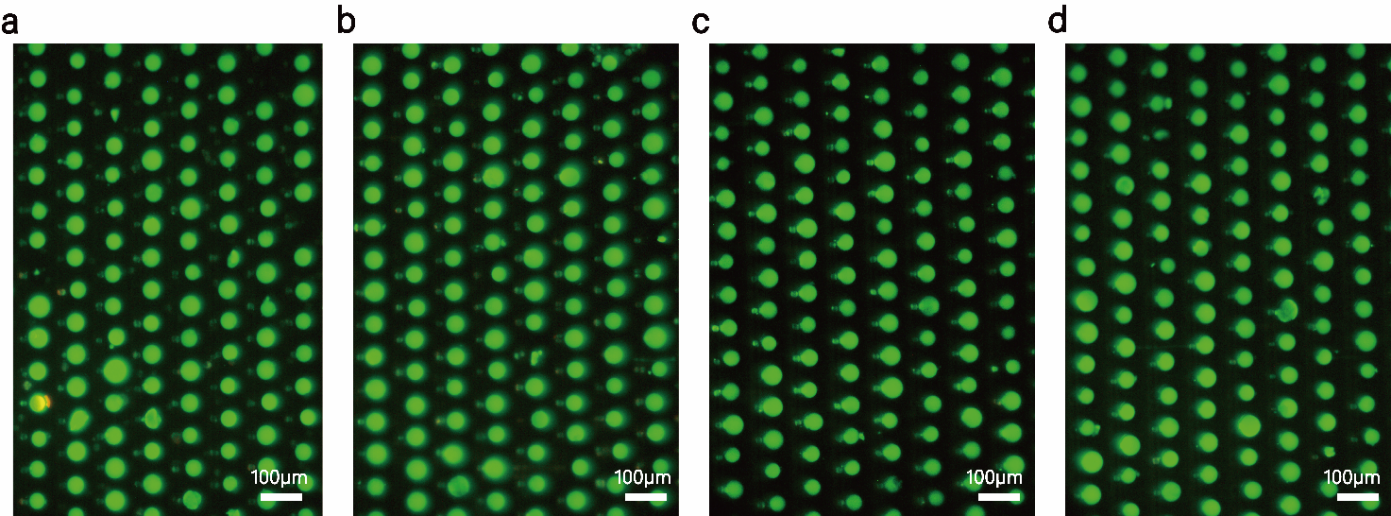


**Figure S3.** Single-cell pairing rate at different flow rates after 6 minutes of sample loading. (a) 2 μL/min flow. (b) 4 μL/min flow. (c) 6 μL/min flow rate. (d)8 μL/min flow rate.

**
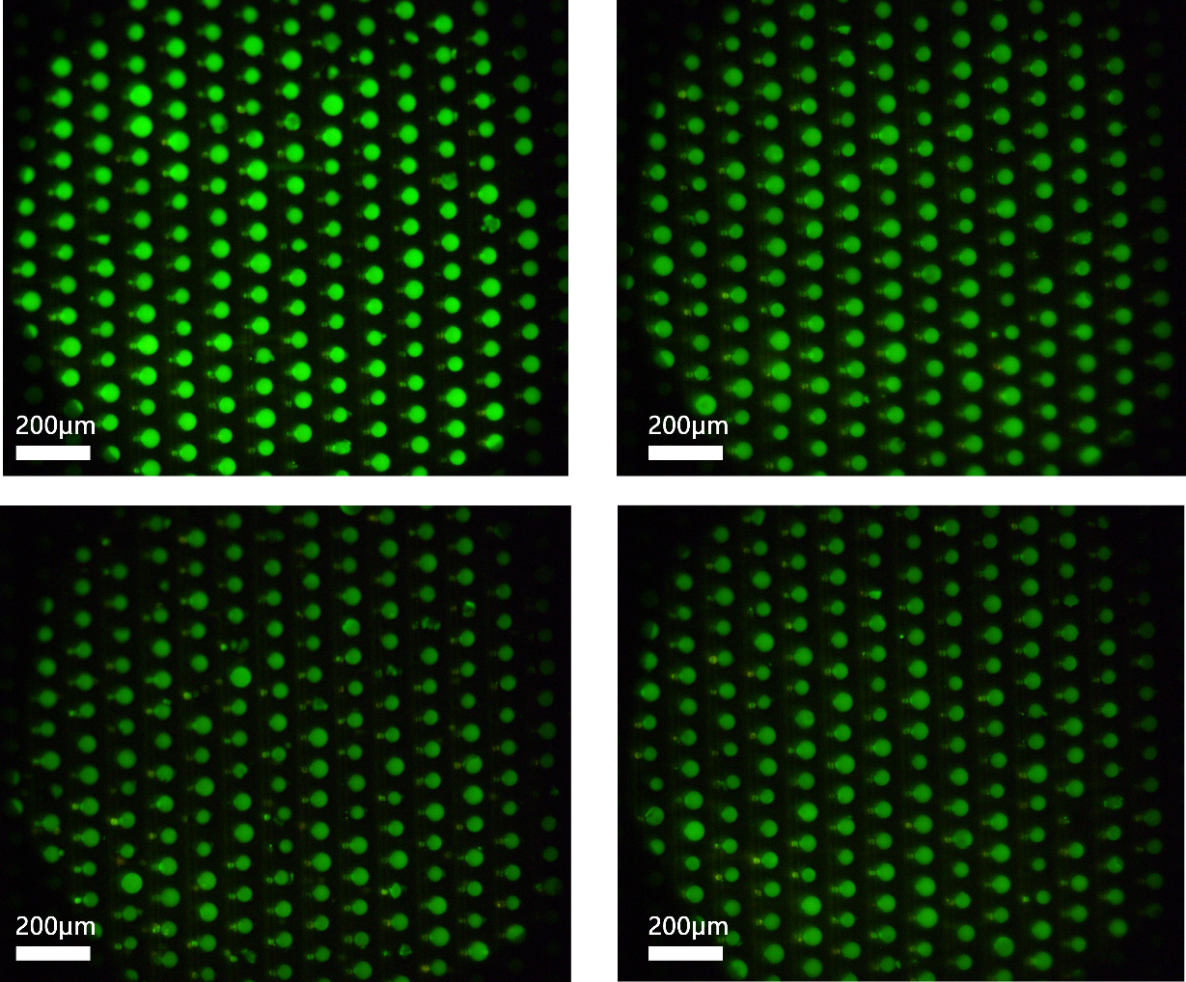
Fugure S4.** Fluorescent images of approximately 800 ddNA units taken after DEP-assisted cell capture under 6 μL/min flow rate, demonstrating over 75% single cell pairing rate. The imaging areas were randomly selected from different sectors.


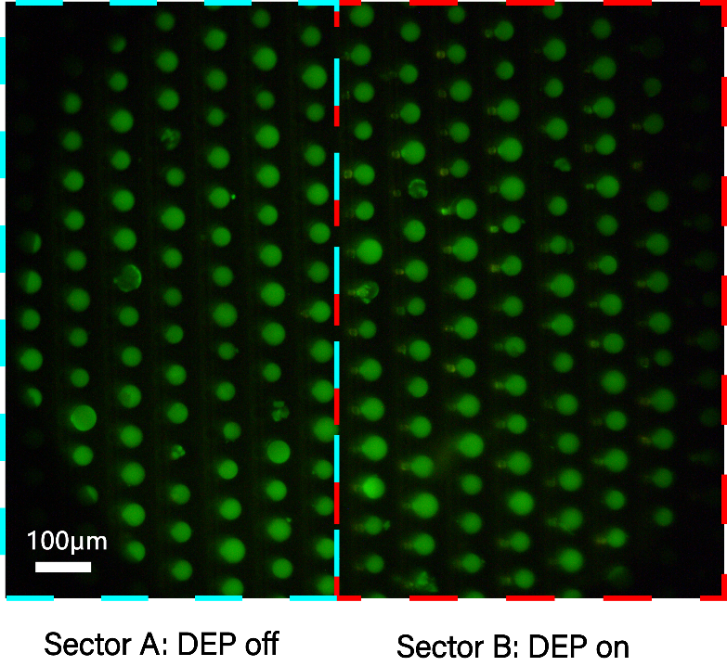


**Figure S5.** Comparison of the IDE sectors activated and not activated by the DEP signal on one device. The left sector (dash cyan frame) was not activated by the DEP signal, where almost no cells were captured. The right sector was activated by the DEP signal, where most ddNA units have cells paired with beads.


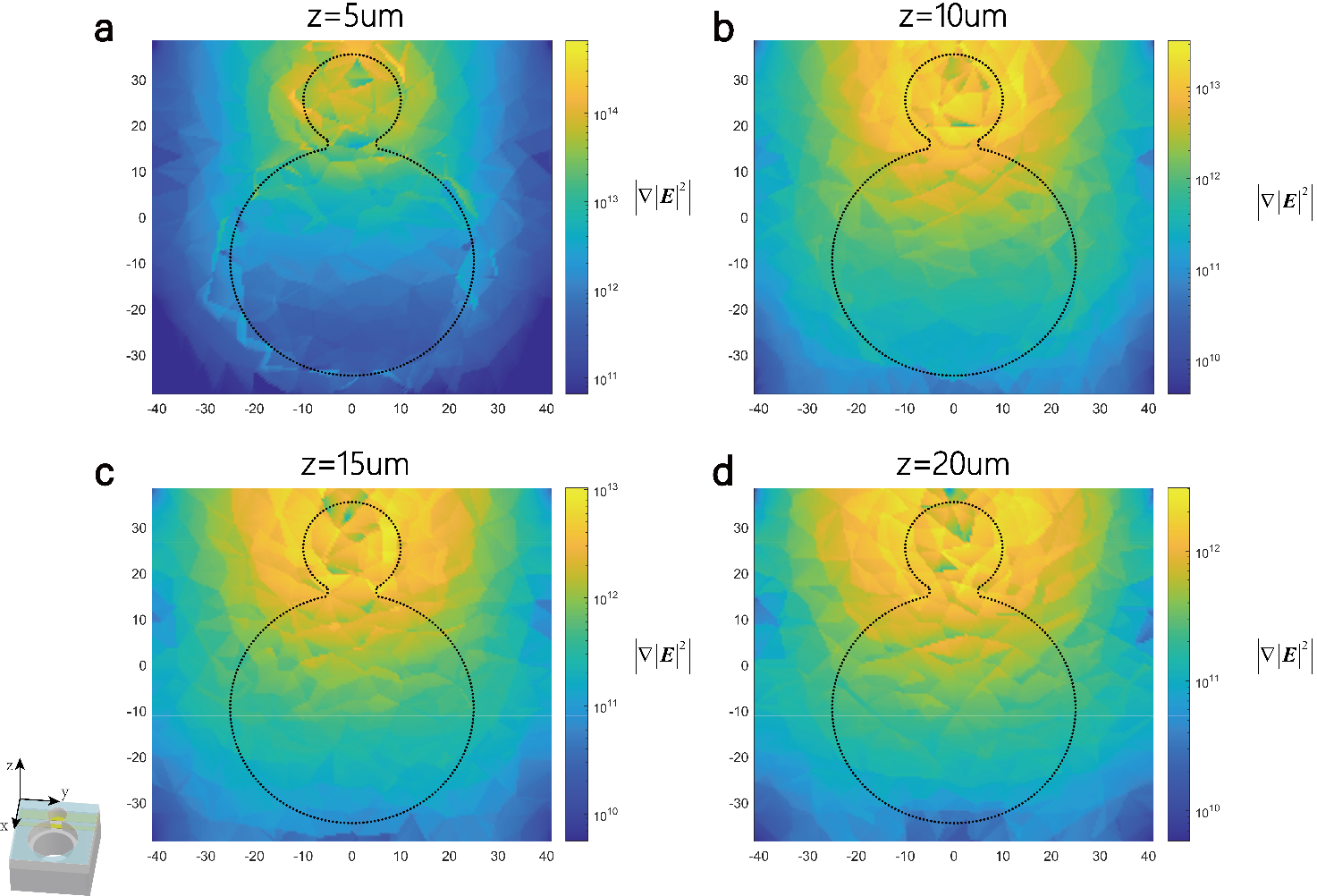


**Figure S6.** COMSOL Simulations. Color maps show the distribution of the magnitude of the $\nabla{|\boldsymbol{E}|}^{2}$ at different heights above one ddNA unit. (a) z = 5 μm. (b) z = 10 μm. (c) z = 15 μm. (d) z = 20 μm above.


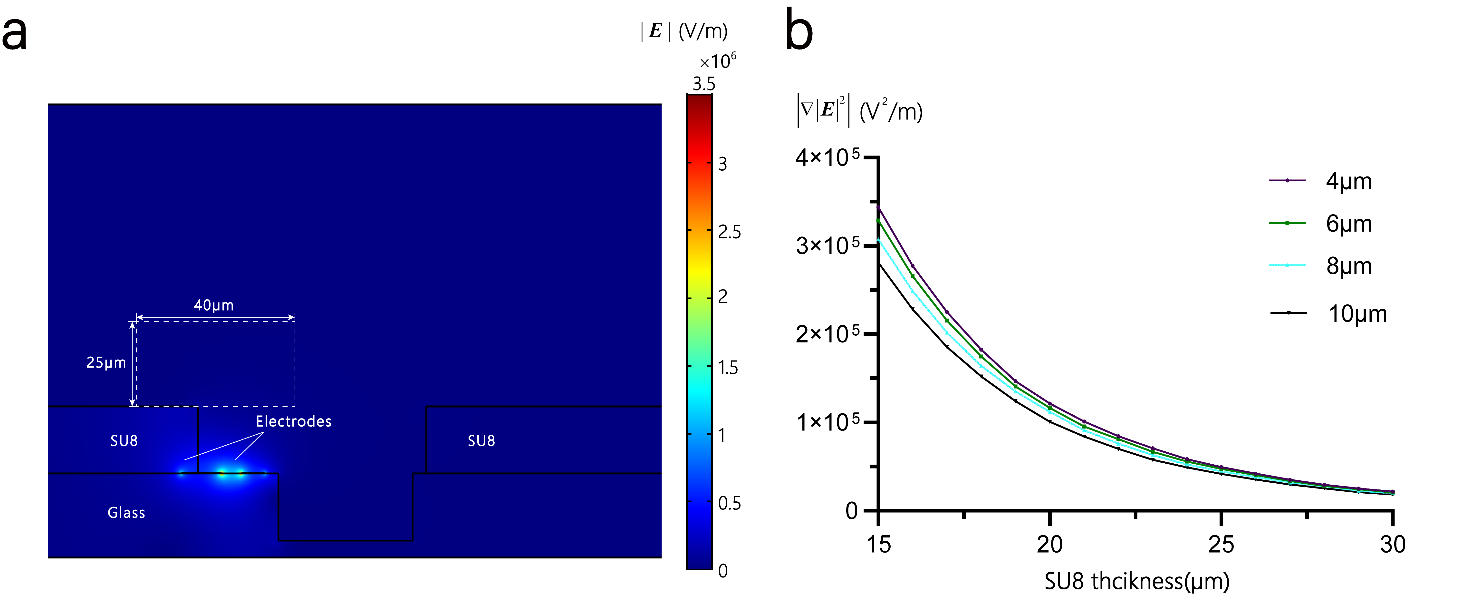


**Figure S7.** COMSOL Simulation results for different IDE finger gaps and SU8 thickness. (a) Simulation domain and color map of the electric field intensity. The white dashed rectangle above the electrode represents the integration region of the magnitude of the electric field gradient $(\nabla{|\boldsymbol{E}|}^{2})$. (b) The integrated magnitude of the electric field gradient for different IDE finger gaps and SU8 thicknesses. IDE finger gap does not significantly influence the electric field gradient. The voltage applied in all simulations is the same as the experimental voltage (8 Vpp, 1 MHz).

**
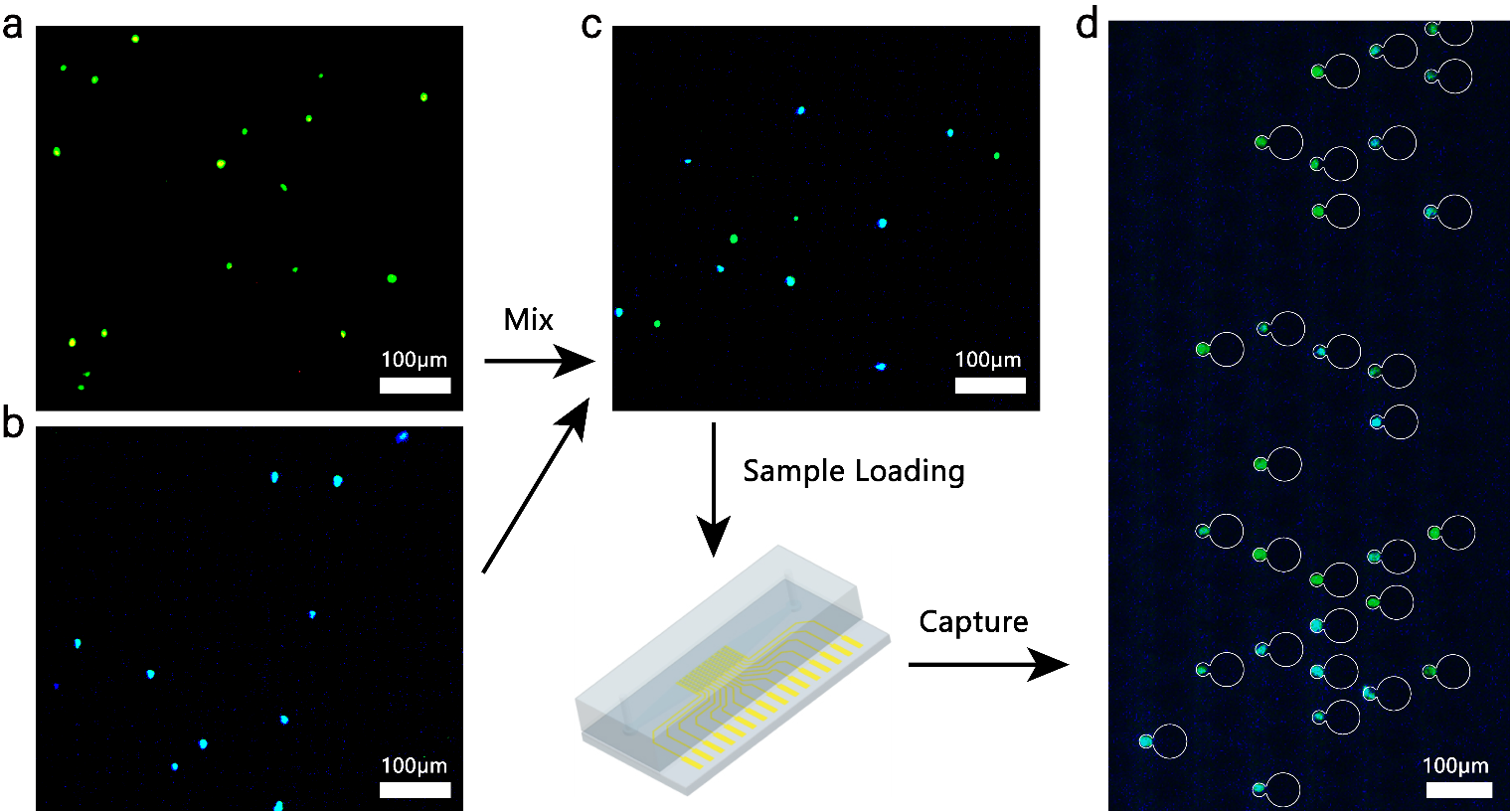
**

**Figure S8.** Mixed sample capture. (a) Microscope image of HEK-293 cells stained with green fluorescent dye. (b) Microscope image of NIH-3T3 cells stained with blue fluorescent dye. (c) Merged image of the mixed cell sample. (d) Merged image of the mixed cell sample capture with DEP force. The ddNA chip allows the compartmentalization of different cells into different ddNA units, which is a fundamental function for microfluidic single-cell platforms.


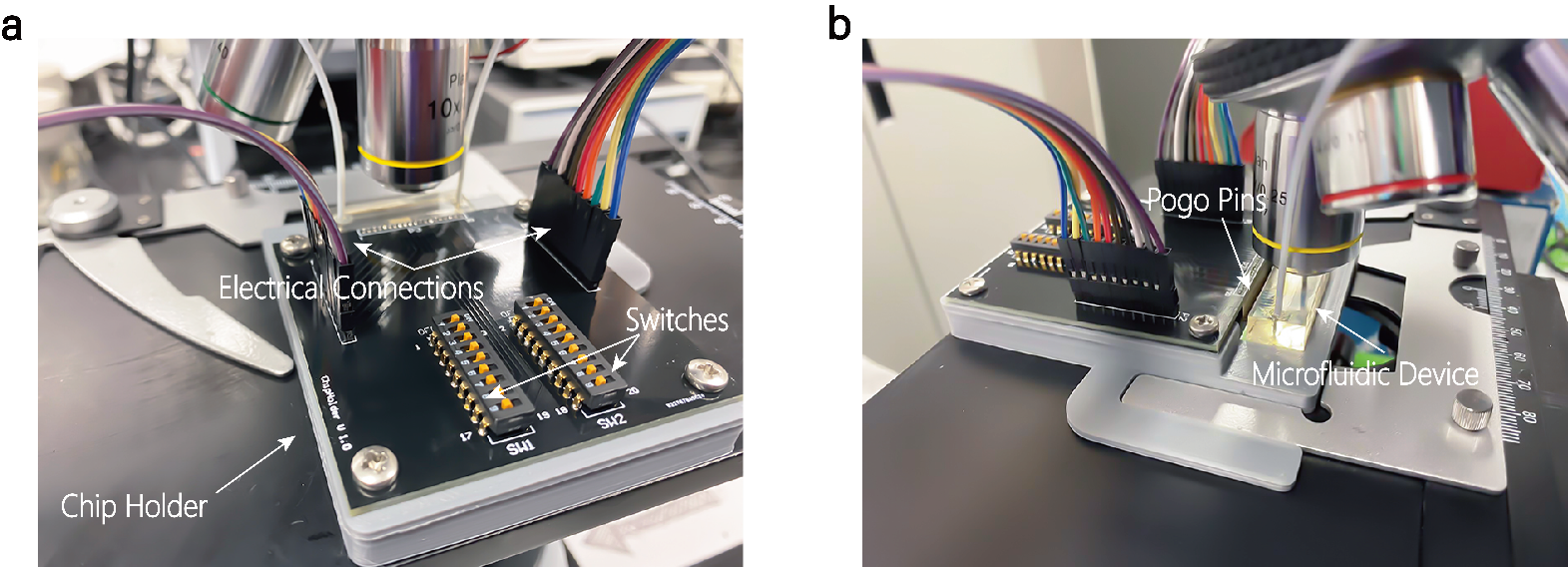


**Figure S9.** Custom-made chip holder. (a) The front-side view of the chip holder shows the switches used to route signals to the selected sectors. (b) The backside of the chip holder shows the pogo pins used to electrically connect the pads of the microfluidic device to the respective instrumentation.
